# Reconstructing Taiwan’s land cover changes between 1904 and 2015 from historical maps and satellite images

**DOI:** 10.1038/s41598-019-40063-1

**Published:** 2019-03-06

**Authors:** Yi-Ying Chen, Wei Huang, Wei-Hong Wang, Jehn-Yih Juang, Jing-Shan Hong, Tomomichi Kato, Sebastiaan Luyssaert

**Affiliations:** 1 Research Center for Environmental Changes (RCEC), Academia Sinica, Taipei, 11529 Taiwan; 2 Department of Geography, National Taiwan University, Taipei, 10617 Taiwan; 3 Central Weather Bureau, Taipei, 10048 Taiwan; 4 Research Faculty of Agriculture, Hokkaido University, Sapporo, 060-8589 Japan; 5 Department of Ecological Sciences, Vrije Universiteit Amsterdam, Amsterdam, 1081 HV The Netherlands

## Abstract

A new reconstruction of changes in Taiwan’s land cover and estimated uncertainty between 1904 and 2015 is presented. The reconstruction is made by integrating geographical information from historical maps and SPOT satellite images, to obtain spatially explicit land cover maps with a resolution of 500 × 500 m and distinguishes six land cover classes: forests, grasslands, agricultural land, inland water, built-up land, and bare soil. The temporal resolution is unbalanced being derived from four historical maps describing the land cover between 1904 and 1994 and five mosaic satellite images describing the land cover between 1995 and 2015. The uncertainty of the historical maps is quantified to show the aggregation error whereas the uncertainty of the satellite images is quantified as classification error. Since 1904, Taiwan, as a developing country, has gone through a not unusual sequence of population growth and subsequent urbanization, a decoupling of the demand for agricultural land from population growth, and a transition from shrinking in forest area to forest expansion. This new land cover reconstruction is expected to contribute to future revisions of global land cover reconstructions as well as to studies of (gross) land cover changes, the carbon budget, regional climate, urban heat islands, and air and water pollution at the national and sub-national level.

## Introduction

Land cover has been defined as “the attributes of the Earth’s land surface and immediate subsurface, including biota, soil, topography, surface and groundwater, and human (mainly built-up) structures”^1^. In this study, land cover changes are defined as a shift from one land cover class, such as a specific type of vegetation cover, to another. Land cover change can occur due to the expansion of croplands, deforestation, or a change in the extent of urban area^1^. Such changes can occur naturally, as has happened throughout the Earth’s history when the vegetation cover has adjusted to geological and climatic changes, or it can be an indirect result of anthropogenic disturbances, such as human-induced climate change. Given the relatively short time frame of this study, i.e., just over a century, the documented land cover change is expected to primarily be the outcome of direct anthropogenic land cover changes which are driven by changes in land use, the “purpose for which humans exploit the land cover”^1^. In this study land cover changes include changes between forest, grassland, cropland, inland water, bare soil, and infrastructure including housing.

Historical maps are the primary source of land cover information^2–7^. The oldest known topographic map describing Taiwan is the “Map of Tamsuy and nearby villages including the island Kelang” (originally called the “Kaartje van Tamsuy en omleggende dorpen, zoo mede het eilandje Kelang”) made by Dutch colonizers and shows the location of aboriginal tribes and the distribution of vegetation in 1654^8^. Although this and similar maps give some idea of the human population distribution during that time, their spatial coverage is very limited and the scale is inaccurate. In later decades, the gold mining regions were mapped^9,10^, but these maps are difficult to merge with modern day geographic information. From the late 1600s to the early 1700s, Taiwan was divided into Dutch, Spanish and Chinese sectors, each being occupied by different military powers. During that period, military maps provide a detailed description of the surroundings of the most important fortresses such as those at Zeelandia (Tainan), Taipei, and Keelung. In 1714, the government of the Qing Empire used triangulation to complete a map of the six administrative units in western Taiwan it controlled at that time. In 1904, the then Japanese ruler published another military fortress map of Taiwan. In contrast to previous maps, they applied survey techniques. This and its coverage (i.e., ∼60% of the island including all lowlands) make it the oldest map that can be used for land cover reconstruction. Since then accurate topographic maps have been periodically published by the consecutive Japanese, Chinese and Taiwanese governments, resulting in reliable spatial information about Taiwan.

Already in the 1990s, the International Geosphere-Biosphere Program (IGBP) had already organized several international conferences to initiate collaborative efforts to reconstruct land cover changes across Eastern Asia. For Taiwan, this effort resulted in a century long history of land cover reconstruction of the area surrounding cities such as Yilan and Taipei^11–13^. Subsequent (international) projects created century to millennia-long spatially explicit global land use or land cover reconstructions, including for Taiwan^14–18^. Nevertheless, even the global reconstructions with the finest spatial resolution are considered too coarse for an island the size of Taiwan (i.e., 36.2 10^3^ *km*^2^) because global reconstructions are often based on assumptions that are questionable at finer resolutions, for example, the assumed long-term average regional deforestation rate and/or a mean growth rate of the human population^19–22^. Consequently, for regions where assumptions are made but data lacking, the resulting global reconstructions may have a low credibility for regional applications^23^. The spatial distribution of the grasslands in Land Use Harmonization (LUH2), for example, differs from the spatial distribution found on historical maps of Taiwan (Fig. S1). Furthermore, notwithstanding the observed increase in forest cover over the past 35 years^24^, some global reconstructions suggest there has been a decrease in Taiwan’s forest cover from 50% in 1975 to 45% in 2010^25–27^.

Since the Academia Sinica established its dedicated Center for Geographic Information Systems in 2003^28^, the center, among other tasks, has collected and digitized historical maps of Taiwan. Unlike the 1990s, when the IGBP initiated their land cover reconstruction initiative, historical maps containing land cover information of the entire island and spanning over a century in time are now available. However the fact that these maps were written in Japanese or Chinese, has likely hampered their inclusion in the global reconstruction efforts led by European and North American researchers^14,16,18^. Following in their footsteps and other regional studies^7,29–31^ the objective of this study is to use historical maps and satellite images as the basis for the first high resolution (500 × 500 m), century-long, wall-to-wall reconstruction of land cover changes in Taiwan. The spatial and temporal extent of this objective differs from previous studies^11–13,32^ which were limited to a specific city or region. The outcome of this effort is expected to contribute to future revisions of global land cover reconstructions (see Table 1 in Fuchs *et al*.^7^) as well as to studies of (gross) land cover changes, the carbon budget, regional climate, urban heat islands, and air and water pollution at the national and sub-national scale.

**Table 1:** Absolute loss (*km*^2^), absolute gain (*km*^2^), net change (*km*^2^), and annual change rate (*km*^2^ *y*^−1^) for all land cover types for different periods between 1904 and 2015.

		1904–1926	1926–1956	1956–1982	1982–1994	1994–2000	2000–2005	2005–2010	2010–2015	annual change rate
Forests	gain	4,034	1,050	3,671	5,091	1,312	1,712	2,265	1,968	
	loss	1,007	3,671	4,133	1,041	1,853	1,375	1,660	2,639	
	gross	5,041	4,721	7,804	6,132	3,165	3,087	3,925	4,607	347
	net	3,026	−2,621	−462	4,051	−541	337	605	−672	34
	rate	138	−87	−18	338	−90	67	121	−134	
Agricultural	gain	2,447	3,322	4,231	1,724	2,833	2,472	1,931	2,325	
	loss	1,863	1,590	4,568	4,795	1,861	2,692	3,306	2,253	
	gross	4,310	4,912	8,799	6,519	4,694	5,164	5,237	4,578	398
	net	585	1,732	−337	−3,071	973	−220	−1,375	72	−15
	rate	27	79	−15	140	44	−10	−62	3	
Grassland	gain	361	261	710	705	457	297	943	1,548	
	loss	3,545	1,390	999	711	743	475	298	845	
	gross	3,906	1,651	1,708	1,415	1,200	772	1,241	2,393	129
	net	−3,138	−1,130	−289	−6	−286	−178	644	703	−34
	rate	−145	−38	−11	−1	−48	−36	129	141	
Built−up	gain	98	82	1,621	762	1,181	1,268	1,335	1,462	
	loss	107	94	93	65	1,006	1,230	1,306	1,033	
	gross	205	176	1,715	827	2,186	2,498	2,641	2,495	115
	net	−9	−11	1,528	697	175	38	29	430	26
	rate	0	0	59	58	31	8	6	86	
Bare soil	gain	231	116	841	307	153	177	293	177	
	loss	116	191	439	887	170	165	178	290	
	gross	346	306	1,280	1,193	322	342	471	467	43
	net	115	−75	403	−580	−17	12	115	−113	−1
	rate	5	−3	15	−48	−3	2	23	−23	
Inland water	gain	255	2,351	1,314	335	432	631	548	293	
	loss	765	163	2,111	1,254	650	558	517	630	
	gross	1,020	2,514	3,425	1,589	1,082	1,189	1,065	923	115
	net	−510	2,188	−797	−919	−219	74	32	−337	−4
	rate	−23	73	−31	−77	−36	15	6	−67	

## Results

### Temporal changes in land cover

Given the relatively high spatial resolution of the information that was used in this reconstruction, i.e., 500 × 500 m, net changes as well as bi-directional changes could be analyzed. Although the temporal resolution of the available data sources is unbalanced, the longest period between two observations is less than 30 years. Hence, bi-directional changes between observations could be considered to be marginal, which justifies calling these bi-directional changes gross land cover changes.

**Figure 1:**
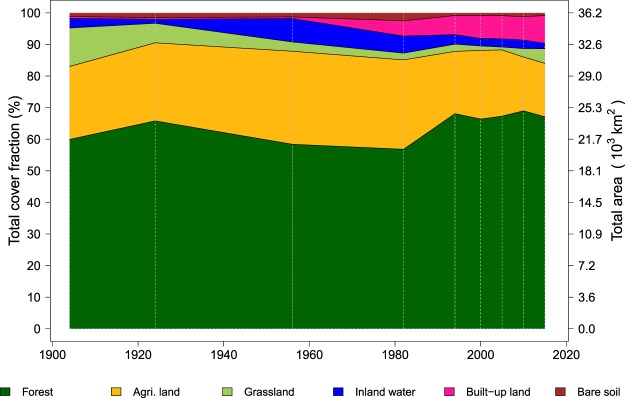
Temporal dynamics of the net land cover changes in Taiwan between 1904 and 2015 based on historical maps, remote sensing images and forest inventory statistics.

**Figure 2:**
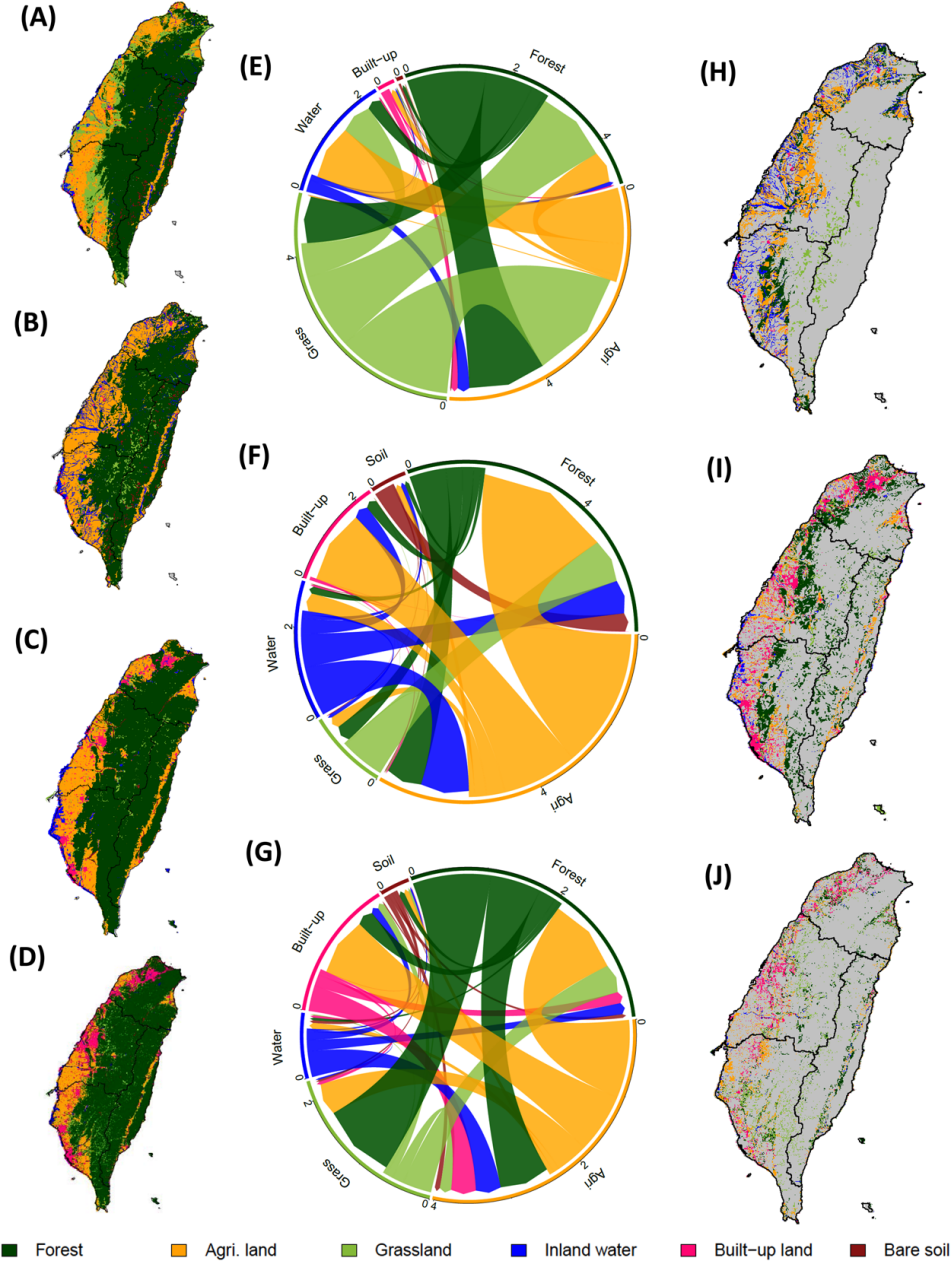
Temporal and spatial gross land cover changes. Reconstructed land cover maps for the years 1904, 1956, 1994, and 2015 (**A**–**D**). Gross land cover changes in Taiwan expressed in 10^3^ *km*^2^ among the three selected periods (**E**–**G**). The direction of the arrows in the chord diagram indicates a change from the original land cover class to a new land cover class. The size of the arrows represents the land cover change area. Spatial distribution of land cover changes in 1956, 1994 and 2015 (**H**–**J**). The black solid lines shows the border of four water resources sectors in Taiwan.

The reconstruction covers three periods which coincide with different stages of urbanization in Taiwan. The first period, from 1904 to 1950, roughly delimits the period of Japanese colonization, the Chinese civil war and post-war reconstruction with a stable portion of land being used for urbanization. Land cover during this period is described by the 1904 and 1926 maps. In 1904, the first year of the reconstruction, the top three land cover classes were forest, agricultural land and grassland, which covered respectively, 60%, 23%, and 12% of the land area in Taiwan (Fig. 1). The first peak in forested land was observed around 1926, covering around 64% of Taiwan. During the first period, the portion of land allocated to infrastructure and bare soil remained at 2% but there were substantial changes in other land cover: e.q., agricultural land increased from 23% in 1904 to 34% in 1956 (Fig. 1). Although the peak in agricultural land use itself was reached in the second period (see below), changes in land cover mainly occurred in the first period. Agricultural expansion was realized into former grasslands and forests located in the lowlands of western Taiwan (Fig. 2A,B,E and H). As a consequence, the grassland cover shrank by about 9% from 12% to 3%. Following the 1956 peak, the land allocated to agriculture started to decrease, a trend that continues until today. Between 1951 and 1995, the portion of built-up land increased sharply (Fig. 1). This is defining feature of the second period which roughly coincides with Kuomintang rule. Maps for 1956, 1982, and 1994 are available for this period. The urban expansion, which occurred in the second period consumed agricultural and forested land in the vicinity of larger cities (Fig. 2B,C,F and I). Except in eastern Taiwan, losses in agricultural land were mostly compensated for by converting low altitude forests into agricultural land. The loss of forest was in turn compensated by the afforestation of grasslands. Also, the interchange between forested and agricultural land was substantial.

In the third period, from 1996 to 2015, 10% of the land was built-up but the growth in urbanization slowed down compared to the previous period (Fig. 1). The third period is documented by remote sensing images for 2000, 2005, 2010, and 2015 and roughly coincides with Taiwanese rule. In this period, forested land gradually recovered to reach a peak at 67% in 2010, an increase of 10% since 1982. Because there was very little grassland left, afforestation during the third period took place on former agricultural land (Fig. 2C,D,G and J). During the third period, urban expansion took place on former agricultural land and forests.

Note that in all three periods, a small fraction of built-up land was apparently converted into forest, grassland or croplands (Fig. 2E–G). Although some of these changes may relate to forest recovery under watershed management in upland areas or agricultural expansion in southern Taiwan, the majority of these changes should be interpreted as classification error.

### Gross land cover changes

The gross change, net change, gain and loss for every land type, over all eight reconstruction periods are calculated and presented in Table 1. The long-term gross change rate is about one order of magnitude higher than the net change rate during the whole reconstructed period. Land types such as forested or agricultural land show a similar long-term gross change rate of 347 *km*^2^ *yr*^−1^ and 398 *km*^2^ *yr*^−1^, respectively. The long-term gross change rate for grasslands, built-up land and inland water are similar and could amount up to 129 *km*^2^ *yr*^−1^. Bare soil has a long-term gross change rate of about 40 *km*^2^ *yr*^−1^. The details of the gross land cover changes for each period are presented in Table S2.

### Spatial distribution of land cover change

**Figure 3:**
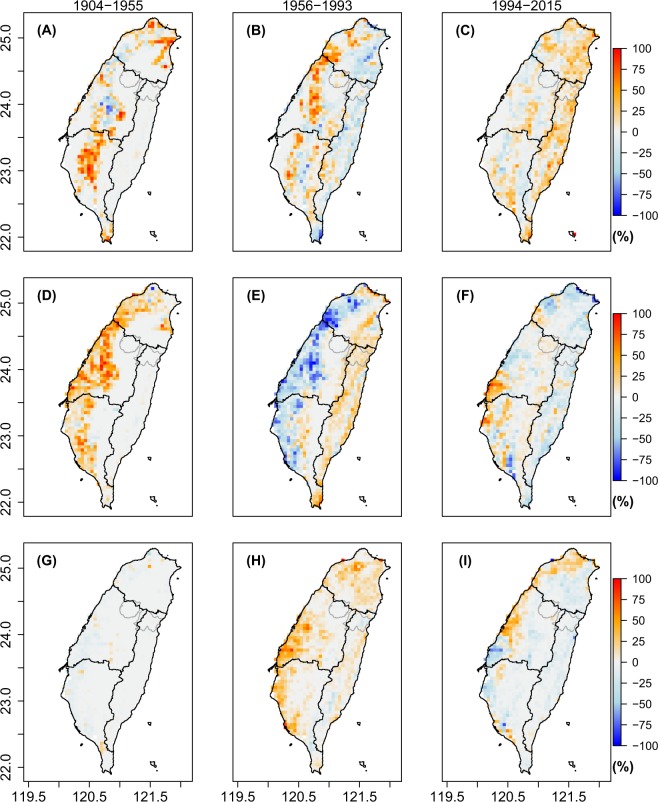
Spatial distribution of land over changes for forests (**A** to **C**), agricultural land (**D** to **F**), and built-up land (**G** to **I**) in Taiwan for three distinct periods: 1904–1955 (**A**,**D**,**G**), 1956–1993 (**B**,**E**,**F**), and 1994–2015 (**C**,**F**,**I**). Blue indicates a decreasing fraction and red an increasing fraction. The black lines show the border of the four water resource districts and the gray lines show the borders of Shei-Pa and Taroko National Parks.

The spatial analysis distinguished the same three time periods as found in the temporal analysis. In comparison with the total forest area (60%), the net change in forest cover (±5%) was relatively small, but the spatial dynamics across the three periods was considerable. In the first period from 1904 to 1945, the forest cover fraction increased in both the northern and southern regions, meanwhile the forest area decreased in the central region (Fig. 3A). For the time period from 1946 to 1993, the forest cover recovered in central Taiwan but at the same time decreased in the high mountain areas and parts of eastern Taiwan (Fig. 3B). For the third time period, from 1994 to 2015, forest cover gradually recovered in all regions (Fig. 3C, Table 1), while some areas presented a slight decrease in the forest cover fraction. Note that our analysis shows no major land cover changes since 1904 in the Shei-Pa and Taroko National Parks, confirming their protected status.

At the beginning of the second time period, between 1949 and 1956, the share of agricultural land increased by 14% a change that was largely confined to the central region. The majority of the new agricultural land was created by conversion of grasslands and forests (Fig. 3D). Towards the end of the second period, agricultural expansion in the central region changed to growth in forests and built-up land. Agricultural activity moved towards the eastern region where forests were converted into agricultural land (Figs 3E and 3H). During the third period from 1994 to 2015, the overall share of agricultural land decreased except for the border region between the central and southern regions (Fig. 3F).

In the first time period, the increase in built-up land was restricted to Taipei in the north and Pingtung in the south (Fig. 3G). During the second period, from 1956  to 1993, the suburban areas of cities such as Taipei, Taichung, Changhua, Chiai, and Tainan, expanded by almost 2,500 *km*^2^ (Fig. 3H; Table 1). During the third period, from 1994 to 2015, the expansion of built-up land continued but slowed down overall, with a few exceptions, such as around Taichung, Taoyuan and Keelung (Fig. 3I). Our analysis hints at a decrease in built-up area in the region between the border of the southern and the central regions. We could find no evidence in support of such a decrease but noticed that the location and pattern of the decrease coincides with the 2.0 km wide riverbed of the Zhuoshui River which has strong seasonal dynamics. The apparent decrease in built-up land is, therefore, thought to be a classification error, unique to this area of Taiwan.

### Uncertainty of the land cover reconstruction

**Figure 4:**
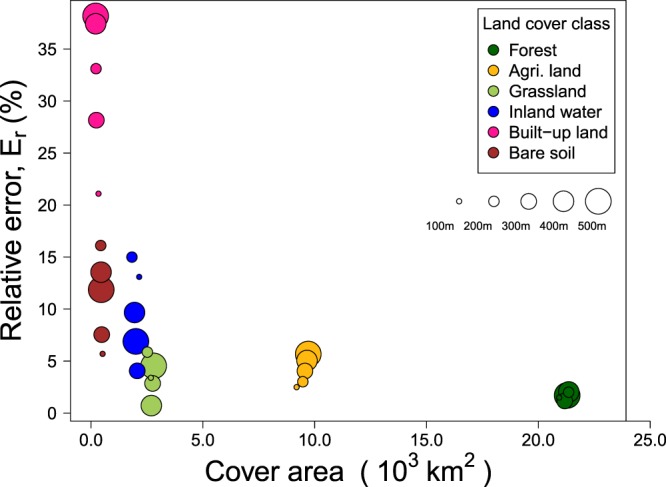
Estimated uncertainty of land cover areas. The uncertainty was quantified as the spatial aggregation error from the majority approach. The circle size represents the aggregated grid spacing from 100 m to 500 m. The values were the average from the years 1904 and 1956.

For historical maps digitized and rasterized at a spatial resolution of 100 × 100 m, the reconstruction was presented using a 500 × 500 m by making use of a majority approach to determine the land cover class of the aggregated pixels. The error introduced by the aggregation is thought to be much larger than the classification errors made at the time of the land survey, the errors made when digitizing the historical maps, the errors from features that are not correctly scaled on maps, e.g., the width of irrigation canals, or the classification errors made when reclassifying the digitized historical maps into our six land cover classes (Table 2). Aggregation errors were calculated for five different spatial resolutions between 100 × 100 m and 500 × 500 m for the 1904 and 1956 map. The average between both maps was reported. The smallest classification error, i.e., 3 to 6%, was found for the most abundant land cover classes, i.e., forest, agricultural lands and grasslands (Fig. 4). Despite the similarity in area cover between grasslands and inland waters, the error for inland waters is almost three times higher than for grasslands.

**Table 2:** Conversion rules applied to different maps.

Map name (published year)	Original land cover type	Attributed land cover type
Taiwan fortress map (1904)	Conifer, Hardwood, Bamboo, Mixed forest	Forest
	Paddy rice field, Dry farm, Tea tree farm	Agricultural land
	Grassland	Grassland
	Water area, Ponds, Sea salt farm, Wetland	Inland water
	Urban	Built-up land
	Riverbed and landslide, Rocks, Graveyards	Bare soil
Taiwan land use map (1926)	Conifer, Hardwood, Bamboo, Mixed forest	Forest
	Paddy rice field, Dry farm, Tea tree farm	Agricultural land
	Grassland	Grassland
	Water area, Ponds, Sea salt farm, Wetland	Inland water
	Urban	Built-up land
	Riverbed and landslide, Rocks, Graveyards	Bare soil
Taiwan forest type map (1956)	Conifer, Conifer hardwood, Hardwood, Conifer plantation, Hardwood plantation, Bamboo	Forest
	Agricultural land	Agricultural land
	Grassland	Grassland
	Water area	Inland water
	Urban	Built-up land
	Riverbed and landslide	Bare soil

The estimated area of built-up land comes with an uncertainty of almost 40%. This large uncertainty is caused by the small cover area in combination with high fragmentation. Until the early 1990s, housing and infrastructure were scattered over the lowlands, especially in the central region^33^ which inflates the aggregation error when a majority approach is used. When objects with a resolution smaller than 100 m, such as houses are scattered in a relatively homogeneous landscape, aggregation by a majority approach will result in an apparent loss of the scattered land use.

Furthermore, the classification results obtained from the SPOT images used in this study was compared against the independent land cover classification retrieved from MODIS for the year 2007 and verified with ground truth data^23^. Image classification was reported to be the major source of errors in the satellite-based land cover maps^34^. Our classification of the SPOT image comes with a classification error of around 30% for inland water and bare soil, and an error below 10% for other land cover classes, such as forested, agricultural land, grassland and built-up land (Table 3). In this study, bare soil and built-up land have similar spectral characteristics which contribute to the misclassification of these two land types. If bare soil and infrastructure are combined into a single land cover type, the classification error drops to around 15% for the combined land cover type. Note that in Taiwan an import source of bare soil and its dynamics movement are landslides in the mountain areas following heavy precipitation events such as typhoons.

**Table 3:** Comparison of the average land cover classification for 2005 and 2010 presented in this study with an earlier classification^23^ for the year 2007.

	Forest	Grassland	Agricultural land	Built-up land	Bare soil	Inland water
This study (*km*^2^)	24.53	0.62	6.84	2.65	0.37	0.99
Cheng *et al*.^23^ (*km*^2^)	23.54	0.65	7.78	3.06	0.25	0.72
Relative error (*E*_*r*_; %)	4.0	4.5	13.7	15.5	32.0	27.0

**Figure 5:**
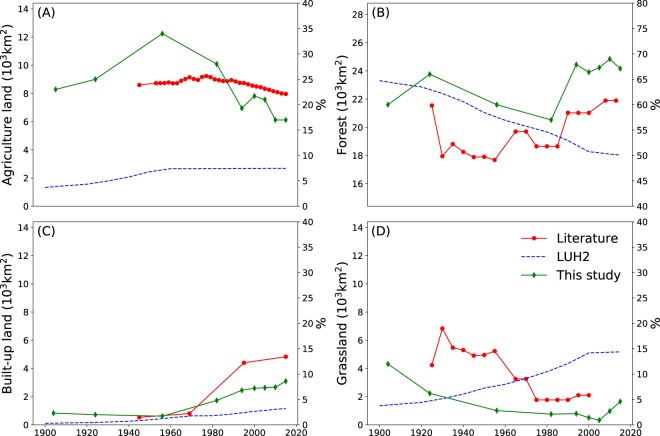
The dynamics of land cover change of Taiwan between 1900 to 2015 from different independent data sources for Forest (**A**), agricultural land (**B**), Built-up land (**C**), and (**D**) Grassland. LUH2 denotes the Land Use Harmonization dataset^35^.

Finally, the temporal trends in the land cover reconstruction were evaluated against a compilation of independent data sources (see Evaluation data). The reconstruction presented in this study largely confirms to the temporal trends derived from independent data sources (Fig. 5). However, the monotonically decreasing trend in forested areas and monotonically increasing trends in grassland and built-up areas illustrated in the LUH2 dataset^16,35^, are at odds with both the reconstruction presented in this study and independent data sources. The absolute differences between our reconstruction, the LUH2 dataset, and the independent data sources are often substantial, i.e., outside the estimated uncertainty intervals.

## Discussion

Changes in built-up land area are closely linked to the expansion of Taiwan’s population. In the early 1900s Taiwan had about 2.5 million inhabitants^36^. Following population growth and the immigration acts that were in force for almost 40 years, the population reached 5.8 million inhabitants in 1945. Then, under the Japanese, more than 72 villages were constructed around the island to accommodate the growing population, but the fraction of built-up land remained below 530 *km*^2^ (or <2% of the total land area). The Chinese rule of Taiwan coincides with a period of rapid population growth. The land area allocated to housing and infrastructure followed suit: from a relatively small coverage of about 800 *km*^2^ in 1969 to 4,400 *km*^2^ in 1995^33^. Since the mid-nineties the population has grown steadily from 21 million to 23 million inhabitants, a growth which is reflected in the built-up area that reached 4,800 *km*^2^ in 2015^37^.

Until 1956, the expansion of agricultural land followed population growth after which population growth and the amount of land used for food production became increasingly decoupled. This decoupling was driven by the green revolution, with a relative improvement of agricultural productivity: for example, brown rice increased from 2.5 *Mg ha*^−1^ in 1960 to 4.5 *Mg ha*^−1^ in the 1990s^38^ due to international breeding programs that resulted in more productive cultivars, automation of farming practices, and the use of synthetic fertilizer and pesticides^39^. Furthermore, large scale irrigation projects that were completed in the late 1960s^32^, the increased use of fishing ponds^40^, as well policies encouraging farmers to become land owners, i.e., the land-to-the-tiller program^41^, have also contributed to the increase in productivity. Recently, some agricultural land has been set aside and is being left fallow due to water shortage and the importation of wheat and other agricultural commodities from overseas^42^.

For over 250 years, agriculture in Taiwan has relied on irrigation ponds to collect rainwater that was then used to irrigate rice paddies and rice terraces. Since 1963, the major agricultural areas have been connected by canals to the Shimen reservoir. The Shimen reservoir and other reservoirs that were constructed later were established at the expense of forested areas. The traditional irrigation system is in danger of vanishing. Nearly 36% of the irrigation ponds present in the year 1993 have been filled to make space for local urbanization or industrialization^43^. The loss in irrigation ponds has not been compensated for by an increase in sweet and salt water fish ponds^40^.

Initially, the Japanese rulers brought their tradition of forest conservation and sustainable management^44^ to Taiwan. The interest of the Japanese in camphor production, from 2,000 to 4,000 *Mg y*^−1^ between 1903 to 1919, is illustrated by a historical report made by the Japanese Forestry Bureau in Taiwan^33^. Despite the population growth and an increase in the land area used for agricultural production, the Japanese afforested grasslands to offset deforestation (see Table 1), meet demands for wood for construction, and sustain forest-based production of commodities, such as camphor and firewood. These policies led to a first peak of forest coverage in 1926^45^ (Fig. 1).

Japanese war efforts in the 1930s and 1940s quickly resulted in net deforestation^46^. The post-war population growth and subsequent urbanization, agricultural expansion, and an increasing land area of infrastructure for irrigation were all realized at the expense of forested areas which reached theirs lowest documented value, i.e., around 51% in 1980^24^. Integrated watershed management, which was a rather new idea in the 1980s, likely triggered a change in attitude towards forest management and forest conservation. This change in attitude was reflected in national laws from the 1990s designed to protect forests within natural reserves, a series of afforestation acts meant to encourage farmers to plant selected tree species on their abandoned agricultural land, a nationwide afforestation campaign in 1996, the Love-Taiwan project in 2008, and the enhanced protection of urban green including street trees. Around the 1980s, the decrease in agricultural land and inland waters freed up more land than required for urbanization, enabling an increase in forest cover. Like many other developed countries^47^, Taiwan has gone through a transition from shrinking to expanding forests.

The absolute differences between the land areas for the different land cover classes obtained in our reconstruction and the land areas derived from independent data sources are often substantial, i.e., outside the estimated uncertainty intervals. When zooming in over Taiwan, the global LUH2 reconstruction completely overlooked the revolution in food production. Starting from around 1960, the trends found by LUH2 have been at odds with the reconstruction and literature sources presented in this study (see Fig. 5 and Table 1). For example, the spatial distribution of grasslands is at odds with the information contained in historical maps (Figure S1). Likewise, global reconstructions may overlook a similar decoupling between population growth and the national demand for agricultural land in countries that have gone through similar socio-economic changes, e.g., Japan, the Republic of Korea (a.k.a. South-Korea) and Vietnam. The discrepancy in temporal trends between global reconstructions on the one hand, and our reconstruction and independent data sources on the other hand, suggests that land reconstructions could benefit from using regional information. Although it is impossible to validate the different reconstructions, i.e., demonstrate that they are an accurate representation of the real system^48^, the spatially explicit character of our reconstruction, its spatial consistency (i.e., the sum of all land cover classes adds up to 100%), and it reliance on historical maps and regional data sources is thought to contribute to its quality.

The discrepancy in the spatial distribution and temporal trends for forested and agricultural land areas between global reconstructions on the one hand and our reconstruction and independent data sources on the other hand, is thought to have far-reaching consequences. Land cover reconstructions are used to drive numerical models. Driving models using global reconstructions rather than the reconstruction presented in this study could lead to an underestimation of carbon storage because forests and agricultural lands have very different carbon stocks and fluxes. Furthermore, models driven by global reconstructions may not match forest inventory data, because the simulated forest will be younger than the observed forests. Likewise, differences in land cover and land cover change propagate in the calculation of surface albedo, surface roughness and evapotranspiration, resulting in difficulties in accurately simulating local weather systems in Taiwan such as the movement of onshore breezes^23^. Furthermore, underestimation of the area built-up land can significantly impact the magnitude of diurnal temperature variation^49^. We, therefore, expect that our land cover reconstruction can make a significant contribution efforts to compile better global land cover reconstructions as well as more accurate regional simulation studies.

## Methods

### Data

#### Historical maps

Since it was established in 2003, the Center for Geographic Information Science, a thematic center of the Research Center for Humanities and Social Sciences within the Academia Sinica has collected, digitized, archived, and shared historical maps of Taiwan of which the oldest map dates back to 1897. The archive currently contains more than 26 historical maps, although only three out of these covers the entire island and contain information on the vegetation cover: (i) the topographic map of 1926, (ii) the land use and forest type map of 1956, and (iii) the agricultural land and land use map from around 1982. In addition to these three maps, the military fortress map of 1904 was also used. It describes the vegetation cover but only for the lowland areas (below ∼600 m above sea level) and thus contains no information about upland areas (between 600 m and 3400 m) which represent ∼43% of Taiwan. For the selected four maps various attributes such as farm, city, grassland, water and forest to mention a few, were digitized as spatially distributed polygons. Spectral analysis was used to extract and digitize features and land types from the coloured map. Black and white maps, however, had to be digitized by hand according to the legend.

#### Forest inventory maps

Starting in 1954, approximately every 18 years, the Forestry Bureau of Taiwan has published the  results of their forest inventory. At present the results of the 1954, 1972, 1990, and 2008 inventories are available. Of these, only the 2008 inventory is available as spatially distributed polygons and therefore qualified to be used in this study. Hence, the 1954, 1972, and 1990 forest inventories were not used in this study.

#### Satellite images

The Center for Space and Remote Sensing Research (CSRSR) in Taiwan holds, among other data, the SPOT 4, 5, and 6 (Satellites Pour l’Observation de la Terre) level 3 products. The level 3 products contain raw reflectance data but the images have been corrected for satellite orbital error, sensor relative error, radiometric error, geometric error and image projection error. The number of missing values was reduced by using mosaic images for Taiwan from the years 1994, 2000, 2005, 2010 and 2015. The subset of selected images used in the mosaic are also listed in Supplementary Table S1. This approach, however, results in a product that cannot be used to study land cover changes within a single year. The first satellite image used in this study was for the year 1994 rather than 1995 which would have resulted in a slightly more balanced temporal resolution.

#### Evaluation data

Century-long and island-wide net land cover change data for Taiwan are available from previous research or can be extracted from global land cover reconstruction datasets, such as LUH2^35,16^. The new historical reconstruction is compared to these largely independent estimates. Independent data on forest cover are available from individual studies^24,46^, and national statistcs^50^; independent agricultural land cover data are obtained from an individual study^33^ and national statistics^51^; an independent estimate of built-up land is obtained from an individual study^33^; and an independent estimate for the grassland area can be obtained from national statistics^50^.

### Preprocessing of the historical maps

**Figure 6:**
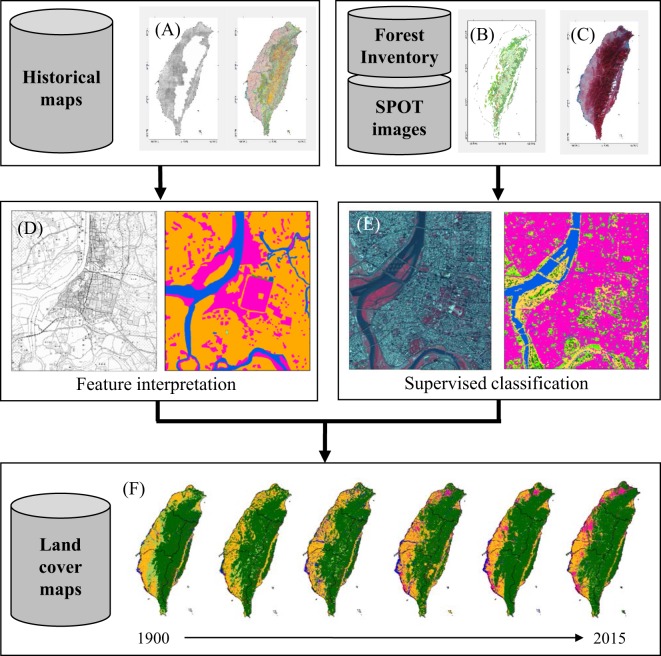
Flow of the data processing applied in this study. Geographical information was collected from different sources such as historical maps (**A**), a forest inventory map (**B**), and satellite data (**C**). Subsequently, historical maps were reclassified into six land cover classes by feature interpretation (**D**). Remote sensing maps were reclassified into six land cover classes by a supervised classification approach that was trained on the forest inventory map (**E**). All land cover maps were finally aggregated to a spatial resolution of 500 × 500 m with six major land cover classes (**F**).

The original attributes of the historical maps were reclassified into six major land cover categories: (i) forest, (ii) grassland, (iii) agricultural land, (iv) inland water, (v) built-up land, and (vi) bare soil which were, except for the bare soil class, in line with other reconstructions such as IPCC LULUCF (Land Use, Land-Use Change and Forestry)^16,52^. The rules applied for reclassifying the original land cover categories into the categories used in the reconstruction are shown in Table 2. The military fortress map of 1904 covers only the lowland areas which account for about 57% of Taiwan. The lack of interest by the Japanese rulers in mapping the upland areas was interpreted as an indication that there was little to no economic infrastructure worth defending present in that area at that time. We, therefore, assumed that all upland areas were covered by forests around 1904 (Table 2). Finally, the polygons of all four historical maps used in the reconstruction were converted to a raster format with a 500 × 500 m resolution (Fig. 6A,D and F).

The 1926 land cover map used counties as the basis for its spatial resolution. Consequently, the 1926 map had to be downscaled to the 500 × 500 m resolution to match the 1982 agricultural land and land use map which had the coarse resolution of all maps used in the reconstruction. First, the digitized polygons that represented the counties were rasterized to the 500 × 500 m resolution and the mesh was left empty. Secondly, this was compared with historical land cover maps for 1904 and 1956. Pixels with no change in the land cover within this period were copied into the empty mesh having no land cover information. Thirdly, within each county, the still empty pixels were ranked according to their altitude. Fourthly, the empty pixels were filled as a function of altitude. High altitude pixels were assigned to be forest until the share of forest recorded in the 1926 map was met. The algorithm continued to allocate the grassland, built-up land, bare soil, and croplands until the shares recorded in 1926 were reached. The remaining pixels at the lowest altitude were allocated to inland waters.

### Preprocessing of the forest inventory maps

The forest inventory map for 2008 was classified and rasterized without further preprocessing, at a resolution of 500 × 500 m (Fig. 6B). The 2008 map was solely used to train the supervised classification of the SPOT images from 2008. Both user and producer classification accuracy were calculated by randomly selecting a 2,500 *km*^2^ domain over Taiwan. The user’s classification accuracy for forests is 96%, 79% for agricultural land, 66% for grasslands, 51% for built-up land, 88% for inland water, and 69% for bare soil. The producer’s classification accuracy is 91% for forests, 84% for agricultural land, 75% for grasslands, 55% for built-up land, 81% for inland water, and 60% for bare soil.

### SPOT satellite images

The level 3 products based on SPOT images contain spectral information and thus needed to be classified prior to their usage in the reconstruction (Fig. 6C). The maximum likelihood classifier in ArcGIS which is a supervised classification algorithm, was applied on SPOT 4 and 5 images from 2008 with less than 10% cloud cover for the spectra at 500–590 nm, 610–680 nm, 790–890 nm, and 1580–1750 nm. The classification network was then applied to the SPOT 4 image for 1994 and the classification network was examined against the topographic map (1:25,000) for the same year.

Following training and evaluation, the classification network was applied to the 1994, 2000, 2005, 2010, and 2015 level 3 SPOT images to obtain the six land cover categories used in the reconstruction. Subsequently, the classified high resolution images (20 × 20 m for SPOT 3 decreasing to 6 × 6 m for SPOT 6) were aggregated into maps with a resolution of 500 × 500 m in line with the resolution of the historical maps. When aggregating, we followed^4^ and assigned each pixel to its dominant land cover category. In doing so part of the spatial information was ignored which was then used in the uncertainty analysis. Finally, across the five mosaic SPOT images used, the 2.1% pixels masked by clouds were assigned to the land cover class of their nearest neighbor (Fig. 6C and E).

### Reconstruction of land cover change from 1904 to 2015

At this point in the analysis we had access to four historical maps and five remote sensing maps with the same land cover classes and the same 500 × 500 m resolution (Fig. 6F). The maps were ordered chronologically and linear interpolation between 2 observations was used for the years without observations. This approach resulted in annual net land cover change maps between 1904 and 2015.

### Uncertainty estimation

It was assumed that, for the historical maps, the major source of uncertainty came from applying the majority spatial aggregation approach^53^. The uncertainty introduced by this approach was quantified for five different aggregation levels ranging from 100 × 100 m to 500 × 500 m. The major source of uncertainty in land cover maps based on satellite images comes from the land cover classification^34^. An independently verified land cover classification based on MODIS^23^ was used to estimate the uncertainty of our reconstruction map between 2005 and 2010. Uncertainties were reported as relative error which was calculated as follows:

1 $${{\rm{E}}}_{{\rm{r}}}=|\frac{{{\rm{A}}}_{{\rm{agr}}}-{{\rm{A}}}_{{\rm{org}}}}{{{\rm{A}}}_{{\rm{org}}}}|,$$

where *E*_*r*_ is the relative error introduced by spatial aggregation (%), *A*_*org*_ is the original land cover area (*km*^2^), and *A*_*agr*_ is the land cover area after spatial aggregation (*km*^2^).

## Supplementary information

Supplementary Information File

## Data Availability

After obtaining permission from the Academia Sinica Department of Taiwan History and Culture in Time and Space, the original/historical maps can be accessed through http://thcts.sinica.edu.tw; gis@gate.sinica.edu.tw and http://thcts.sinica.edu.tw/view.php. The SPOT images that were preprocessed for Taiwan, are not public available unless the research is supported by MOST (Ministry of Science and Technology). All nine reconstruction maps can be downloaded from 10.5281/zenodo.1256484. The R-scripts used for analysing the land cover reconstruction and preparing the figures can be downloaded from http://rpubs.com/yiyingchen/.
