# Supplementary material for: Reconstructing Taiwan’s land cover changes between 1904 and 2015 from historical maps and satellite images
Source: Sci Rep. 2019 Mar 6;9:3643. doi: 10.1038/s41598-019-40063-1 (PMC6403323; doi:10.1038/s41598-019-40063-1)
Supplement: Supplementary file 1 — Supplementary Information File [file 41598_2019_40063_MOESM1_ESM.pdf]

Reconstructing Taiwan's land cover changes between 1904 and 2015 from historical maps and satellite images

Yi-Ying Chen1*, Wei Huang1, Wei-Hong Wang1, Jehn-Yih Juang2, Jin-Shan Hong3,
Tomomichi Kato4, and Sebastiaan Luyssaert5
1Research Center for Environmental Changes (RCEC), Academia Sinica, Taipei, 11529, Taiwan
2Department of geography, National Taiwan University, Taipei, 10617, Taiwan
3Meteorological Information Center, Central Weather Bureau, Taipei, 10048, Taiwan
4Faculty of Agriculture, Hokkaido University, Sapporo, 060-8589, Japan
5Department of Ecological Sciences, Vrije Universiteit Amsterdam, Amsterdam, 1081 HV, the Netherlands

This document contains the supplementary tables and figure for "Reconstructing Taiwan's land cover changes between 1900 and 2015 from historical maps and satellite images"

Table S1 Summary of historical maps, satellite images, and forest inventory map used in this study

Data Source	Represent Period	Data Coverage	Data Type	Publisher or map information	Published Year	Note
Taiwan fortress map	1898~1904	lowland area	historical map	Geological Survey Center in Taiwan	1904	
Taiwan forest and wildland maps	1910-1914	lowland area	historical map	Geological Survey Center in Taiwan	1910-1914	No vegetation information (didn't use)
Taiwan land use map	1926	lowland area*	historical map	Geological Survey Center in Taiwan	1926	
Taiwan topographic maps	1921~1928	whole island	historical map	Geological Survey Center in Taiwan	1921-1928	No vegetation information (didn't use)
Taiwan topographic maps	1924~1944, 1946	part of island	historical map	Geological Survey Center in Taiwan	1924~1946	No vegetation information (didn't use)
Taiwan forest type map	1956	whole island	historical map	Taiwan Forestry	1956	
Taiwan agriculture land and land use map	1982-1983	whole island	historical map	Ministry of the Interior and the Council of Agriculture	1982	
SPOT 2 and SPOT 3	1994	60km by 60km subset images	multi band image	1994/01/03 SPOT-2 (120.5E,23.52N)<br>1994/06/23 SPOT-3 (121.5E,23.75N)<br>1994/08/31 SPOT-2 (121.0E,23.50N)<br>1994/11/17 SPOT-3 (121.1E,23.64N)	1994	
SPOT 2 and SPOT 4	2000	60km by 60km subset images	multi band image	2000/04/18 SPOT4 (121.4E,24.00N)<br>2000/03/21 SPOT2 (121.7E,24.00N)<br>2000/10/08 SPOT4 (120.8E,23.55N)<br>2000/04/18 SPOT4 (120.6E,23.97N)<br>2000/06/05 SPOT4 (120.2E,23.50N)<br>2000/06/21 SPOT4 (120.8E,23.70N)<br>2000/08/17 SPOT2 (121.3E,23.30N)	2000	
SPOT 4 and SPOT 5	2005	60km by 60km subset images	multi band image	20050118 SPOT-5 (120.1E,23.50N)<br>20050119 SPOT-5 (121.1E,23.04N)<br>20050119 SPOT-4 (120.8E,23.61N)<br>20050123 SPOT-5 (121.0E,24.40N)<br>20050123 SPOT-5 (120.7E,23.22N)<br>20050215 SPOT-4 (120.8E,23.68N)<br>20050306 SPOT-5 (120.4E,23.73N)<br>20050725 SPOT-4 (120.1E,23.59N)<br>20051106 SPOT-5 (120.1E,23.59N)<br>20051106 SPOT-4 (121.4E,23.77N)	2005	
SPOT 4 and SPOT 5	2010	60km by 60km subset images	multi band image	2010/01/17 SPOT-5 (120.3E,23.63N)<br>2010/02/01 SPOT-5 (120.5E,23.59N)<br>2010/02/10 SPOT-4 (121.1E,23.60N)<br>2010/02/10 SPOT-4 (121.4E,23.55N)<br>2010/02/20 SPOT-4 (121.1E,23.54N)<br>2010/03/06 SPOT-5 (121.1E,23.54N)<br>2010/08/22 SPOT-4 (121.4E,23.74N)<br>2010/09/12 SPOT-4 (120.7E,23.75N)<br>2010/12/10 SPOT-4 (120.9E,23.00N)	2010	
SPOT 5 and SPOT 6	2015	whole island	multi band image		2015	
4th Forest inventory	2008	national forests**	polygon inventory data	Taiwan Forestry	2008	Forests area from broadleaved forest, needle leaf forest and mixed forest, are combined to a single forest class

*: the spatial resolution is in county level and all up-land areas are assumed as forests.
**: multi-spectrum image information from the forest area in the map were selected as the trainning area for supervised image classification

Table S2 Summary of conversion type of land cover change during different reconstruction periods (km2)

From	To	[1904-1926)	[1926-1956)	[1956-1982)	[1982-1994)	[1994-2000)	[2000-2005)	[2005-2010)	[2010-2015)
Forest	Agri	863	2,335	2,786	538	1,305	853	767	996
Forest	Grass	0	231	313	315	232	152	547	1,037
Forest	Water	23	966	353	99	95	116	89	84
Forest	Built-up	4	46	246	49	179	195	143	447
Forest	Soil	118	93	436	40	43	60	115	77
Agri	Forest	1,317	378	1,919	3,867	627	1,218	1,552	963
Agri	Grass	301	28	316	275	181	101	269	351
Agri	Water	143	1,150	892	172	205	393	345	99
Agri	Built-up	66	21	1,150	402	783	903	1,039	789
Agri	Soil	37	13	292	80	65	78	100	52
Grass	Forest	2,207	554	837	412	384	220	180	553
Grass	Agri	1,170	623	108	211	246	197	74	146
Grass	Water	82	197	10	26	18	5	4	10
Grass	Built-up	16	10	5	55	82	34	26	110
Grass	Soil	69	7	40	8	13	20	14	26
Water	Forest	384	29	538	260	126	120	109	179
Water	Agri	315	126	1,225	632	370	308	252	318
Water	Grass	51	1	70	54	4	9	10	21
Water	Built-up	9	6	219	130	125	113	112	99
Water	Soil	7	2	59	180	26	9	35	14
Built-up	Forest	43	10	5	65	123	104	359	168
Built-up	Agri	52	72	19	0	742	990	740	698
Built-up	Grass	7	0	7	0	32	32	82	95
Built-up	Water	5	11	48	0	103	94	97	64
Built-up	Soil	0	1	15	0	7	10	29	9
Soil	Forest	83	79	372	488	52	51	65	106
Soil	Agri	24	83	47	172	86	63	50	84
Soil	Grass	3	2	6	62	9	4	35	45
Soil	Water	3	27	12	39	11	24	14	37
Soil	Built-up	3	0	3	126	12	24	15	19

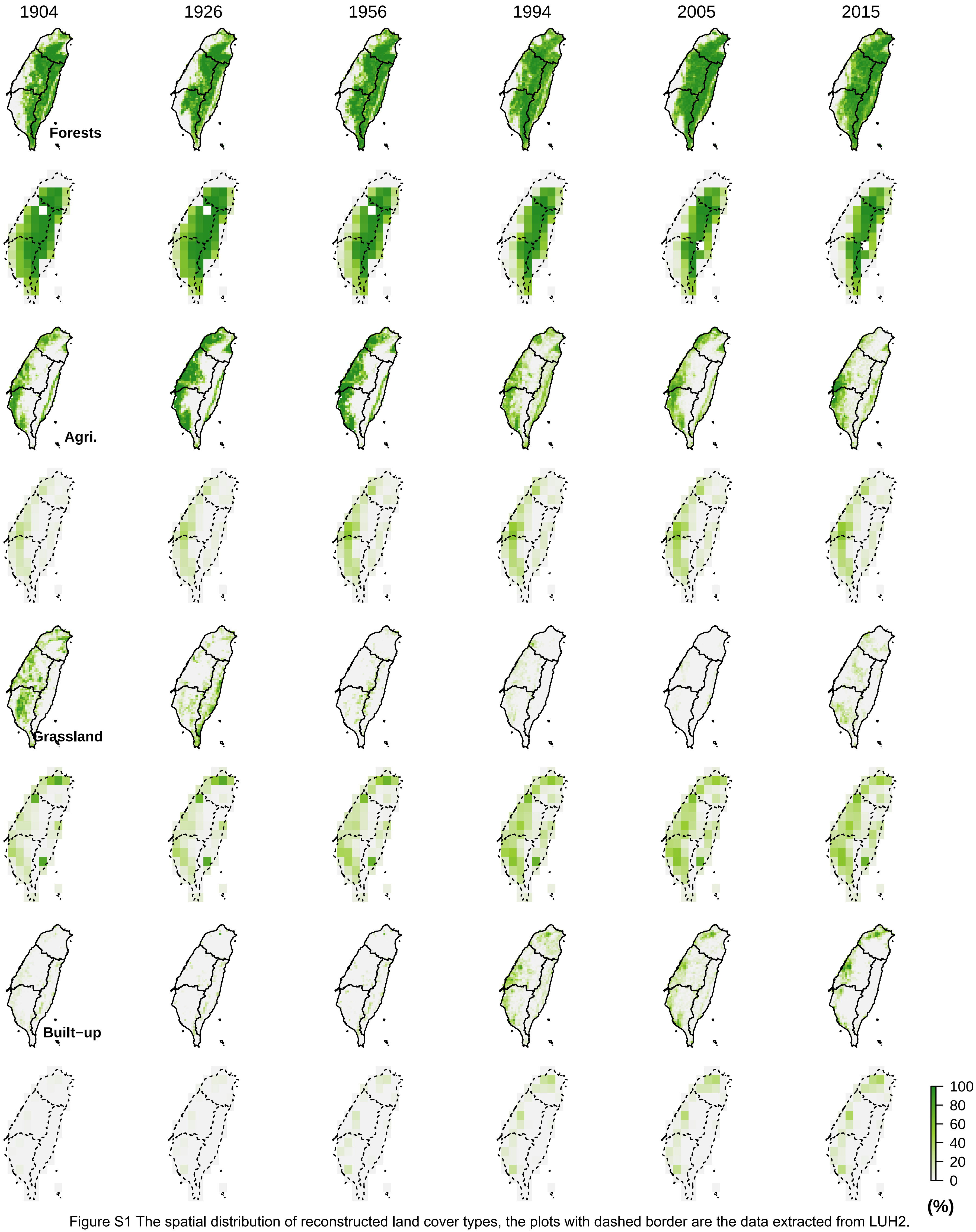

Figure S1 The spatial distribution of reconstructed land cover types, the plots with dashed border are the data extracted from LUH2.
